# Perceptions and experiences of using a nipple shield among parents and staff – an ethnographic study in neonatal units

**DOI:** 10.1186/s12884-016-1183-6

**Published:** 2017-01-03

**Authors:** Renée Flacking, Fiona Dykes

**Affiliations:** 1 School of Education, Health and Social Studies, Dalarna University, Falun, Sweden; 2 Maternal and Infant Nutrition and Nurture Unit (MAINN), School of Community Health and Midwifery, University of Central Lancashire, Preston, UK

**Keywords:** Breastfeeding, Ethnography, Infant, Neonatal care, Nipple shield, Parents, Thematic analysis

## Abstract

**Background:**

Preterm infants have an immature sucking behavior and the capacity to be exclusively breastfed may be reduced for a period of weeks or months, depending on gestational age. Nipple shields have been used, not only as a device to help mothers with sore nipples, but also to facilitate the infant’s latch on to the breast. However, the benefits of using nipple shields have been debated. The aim of this study was to explore perceptions and experiences of using a nipple shield among parents and staff in neonatal units in Sweden and England.

**Methods:**

An ethnographic study was undertaken where observations and interviews were conducted in four neonatal units in Sweden and England. The data were analyzed using a thematic networks analysis.

**Result:**

The global theme was developed and named, ‘Nipple shield in a liminal time’. This comprised of two organizing themes: ‘Relational breastfeeding’ and ‘Progression’. ‘Relational breastfeeding’ was underpinned by the basic themes, ‘good enough breast’, ‘something in between’ and ‘tranquil moment’. ‘Progression’ was underpinned by the basic themes, ‘learning quicker’, ‘short-term solution’ and ‘rescue remedy’. Although breastfeeding was seen primarily as a nutritive transaction, the relational aspects of breastfeeding were of crucial importance. These two organizing themes show the tension between acknowledging the relational aspects of breastfeeding and yet facilitating or supporting the progression of breastfeeding in the period from tube feeding or cup feeding to breastfeeding. It is a liminal time as mothers and their infants are “in between” phases and the outcome, in terms of breastfeeding, is yet to be realized.

**Conclusion:**

This study demonstrates parents’ and staffs’ perceptions of the nipple shield as a short term solution to help initiation of breastfeeding but also as a barrier between the mother and infant. It is important that the mother and baby’s own particular needs are taken into account, in a person-centred way and on an ongoing basis. Furthermore, we need to emphasise the importance of the ‘relational’ whilst understanding the need for ‘progression’. Holding these in balance may be the key to appropriate use of the nipple shield.

## Background

Breast milk mediates unequalled beneficial effects regarding nutritional, immunological and cognitive outcomes in preterm infants (<37 gestational weeks, gw), therefore international recommendations state that infants should be exclusively breastfed for the first 6 months of life [1, 2]. Very preterm infants (<32 gw) constitute a vulnerable population regarding morbidity and mortality [3], they require neonatal care for a substantial period [4] and they constitute a clinically more challenging population on the initiation and sustainability in breastfeeding than more moderately preterm infants (32–36 gw). The more preterm the infant, the more challenging is breastfeeding for the infant. Very preterm infants have a weak oral suction capacity, which may lead to difficulty in getting a sufficient grip on the nipple for nutritive sucking [5]. Although research shows that preterm infants display rooting, efficient areolar grasp, and repeated short sucking bursts from 29 weeks, and occasional long sucking bursts and repeated swallowing from 31 weeks [5], the transition from tube feeding to exclusive breastfeeding at the breast takes time.

Nipple shields are used commonly in many countries and for various reasons, although some are non-evidence based, for example: prevention and treatment for sore or cracked nipples, flat nipples, oversupply, and to facilitate the infant’s attachment to the breast. By using a nipple shield the infant’s palate may be stimulated, which in turn may lead to a more active suction. In a US survey to physicians and allied health professionals specializing in breastfeeding management, the most common reason to use nipple shields among all respondents was to help infants born < 35 gw to latch and nurse [6]. Among researchers and health professionals there are divergent opinions on the benefits and negative aspects of nipple shields [7, 8]. In the preterm population, very few studies have been conducted. Meier et al. [9] reported from a quantitative study where the milk transfer and the duration of breastfeeding were assessed in 34 preterm infants using a nipple shield. Ninety percent of the infants had been provided with a nipple shield because of ineffective attachment to the breast or for falling asleep when attempted breastfeeding. The findings showed that all infants consumed more milk when breastfed with a nipple shield than without. However, there was no association between the duration of nipple shield usage and duration of breastfeeding. In contrast, a prospective survey comprising 1488 preterm infants showed that infants who had used a nipple shield were more than twice as likely to not be breastfed exclusively at discharge compared to those infants who had not been exposed to a nipple shield [10]. Taking the evidence into account, an expert group recently recommended that the usage of an ultra-thin nipple shield may facilitate the preterm infant’s attachment to the breast and milk transfer but that it should only be used after the mother has received qualified breastfeeding support and after substantial trying without a nipple shield [11].

In some Neonatal Units (NUs), nipple shields have been and still are used to support initiation and sustaining of breastfeeding. However, the use of nipple shields is very controversial and study results are contradictory. Furthermore, no study has previously explored the parents’ and staffs’ perspective and experiences of using a nipple shield in NUs. Thus, the aim of the study is to explore perceptions and experiences of using a nipple shield among parents and staff in NUs in Sweden and England.

## Methods

### Study design and setting

An ethnographic study was conducted with an overall aim to explore in-depth the breastfeeding/feeding process in mothers of preterm infants at NUs in Sweden and England [12]. In-depth ethnography was undertaken in two NUs in Sweden and two NUs in England during 2009–2010. England and Sweden were selected due to their differences in terms of culture, context and practices. England, in contrast to Sweden, has a high level of income inequality, a short period of paid parental leave and has a stronger bottle feeding culture. A decreasing trend of breast feeding is occurring in Sweden but compared to England the numbers are still high [13]. The four NUs that were chosen represented different levels of intensive care; one high intensive care unit (e.g. high frequency ventilation and cooling) and one low intensive care unit (e.g. CPAP and ventilation) in each country. The NUs also represented different health care designs and levels of parental involvement.

### Study population

The recruitment of mothers/fathers was based on strategies of maximum variation and purposeful sampling. The latter was utilised in order to obtain data from mothers/fathers who were followed throughout the hospital stay. Theoretical sampling was utilised, in that participants, parents and staff, were selected in order to inform the developing understanding of the breastfeeding/feeding process in mothers of preterm infants at NUs. The only criterion for inclusion was that the infant was born preterm and admitted to the NU. The exclusion criteria were applied to mothers and fathers who experienced temporary or long-term serious medical and mental complications, who did not speak Swedish or English, and who did not wish to participate.

In total, 52 mothers (30 Swedish, 22 English) and 19 fathers (12 Swedish, 7 English) and 102 staff (50 Swedish, 52 English) were observed and interviewed. For the purpose of exploring perceptions and experiences of using a nipple shield among parents and staff in NUs in Sweden and England, all field notes and transcripts of interviews for the whole data collection period (11 months) were read. All data that referred to the subject of nipple shield usage or related comments were identified. Data analyzed derived from observations and interviews from 12 mothers (one from England and 11 from Sweden), three fathers (all Swedish) and 9 staff (three from England and six from Sweden). In this population sample, the infant’s median gestational age at birth was 31 weeks and the mean birth weight was 1599 g. There were three sets of twins and eight mothers were primiparous. The average length of stay in hospital was 51 days.

### Data collection

The study was ethically approved in Sweden by The Regional Ethical Review Board, Uppsala (Dnr 2009/060) and by National Health Service Ethics committee in England. Information (oral and written) about the study was presented to the staff before commencing the study at specific staff meetings. If staff agreed to participate a consent form was provided by the first author of the main study and their written consent obtained. Mothers and fathers were given oral and written information about the study 1 day or more after the infant was admitted to the NICU. In those cases when the infant was critically ill, information was given when s/he had stabilised. All parents, except two mothers, gave written consent that they were willing to participate.

The data was gathered through participant observation [14] and supplemented by interviews. The researcher (first author) took field notes during the observations and the interviews were tape-recorded, when relevant. Out of the 600 h of field work, 300 h were direct observations and interviews. The interviews lasted approximately 45 min but ranged from 10 to 120 min. They were made during day and night shifts for 11 months in total. Some of the mothers were followed during the whole stay and interviewed several times.

### Data analysis

Thematic networks analysis [15] was used to systematize, organize and to describe the findings. Thematic networks analysis was used as a tool in the method for breaking up text and to organize the data. During the analysis interpretation was used to reach a more abstract theme [15]. The material was structured without predetermined themes, an inductive process. The data were read several times to get an overview of the observations and interviews and divided into data from parents and data from staff. The first author read and underlined all meaningful text segments (sentences) that related to nipple shield usage. Text segments were organized into basic themes by a heading. Thereafter, basic themes were merged together into an organizing theme. In the last step, organizing themes were grouped together into global themes. The basic, organizing and global themes were discussed and revised between both authors during the analysis. In this paper we define breastfeeding as feeding from the breast.

## Results

### Context

The four included NUs represented three divergent policies in terms of the usage of nipple shields. In both the English NUs nipple shields were not advocated or used very often and hence parents who wanted to try a nipple shield were required to obtain it by themselves. In both the Swedish NUs, nipple shields were available for free in the units. However, in one of the units there was a proactive use of nipple shields whereas the other unit had a more reactive usage. In England, the Baby Friendly Hospital Initiative’s Ten Steps to Successful Breastfeeding had a strong influence in the NUs. One of the steps (Step Nine) is: “Give no artificial teats or pacifiers (also called dummies or soothers) to breastfeeding infants”. Thus, staff at the English NUs stated that “Nipple shields are banned” or that nipple shields were “discouraged”. In the Swedish NUs, the same influence of the Ten Steps was not seen. In the Swedish context the difference between NUs was more related to an informal policy that a nipple shield is a helpful device in progressing to breastfeeding or that it should be used cautiously and for very good reasons. Even if these were informal policies, the individual nurse conducted her/his own decision making sometimes, taking into account the dyad’s wishes and needs. The only English mother in the study who used a nipple shield had bought it on the Internet because she had “sore and cracked nipples”. Hence, most data from parents are derived from a Swedish context.

**Fig. 1:**
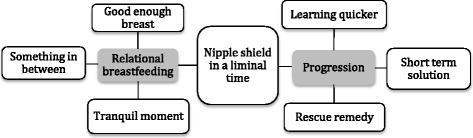
Perceptions and experiences of using a nipple shield among parents and staff—global, organizing and basic themes

The global theme was developed from the data and named, ‘Nipple shield in a liminal time’. This comprised of two organizing themes: ‘Relational breastfeeding’ and ‘Progression’. ‘Relational breastfeeding’ was underpinned by the basic themes, ‘good enough breast’, ‘something in between’ and ‘tranquil moment’. ‘Progression’ was underpinned by the basic themes, ‘learning quicker’, ‘short-term solution’ and ‘rescue remedy’ (see Fig. 1).

### Nipple shield in a liminal time

The global theme was developed, ‘Nipple shield in a liminal time’; comprising of two organizing themes: ‘Relational breastfeeding’ and ‘Progression’. These two organizing themes illustrate the tension between acknowledging the relational aspects of breastfeeding and yet facilitating or supporting the progression of breastfeeding in the period from tube feeding or cup feeding to breastfeeding. It is a liminal time as mothers and their infants are “in between” phases and the outcome, in terms of breastfeeding, is yet to be realized. The themes are now discussed using the narrative and field note data.

### Relational breastfeeding

In Sweden, mothers emphasized that the enjoyment of breastfeeding was facilitated when: the mother could relax and trust herself; when the baby was calm and alert, sucked and swallowed; and when there were few or no disturbances in the breastfeeding session (e.g. weighing before and after, people watching or nurses looking stressed). Although breastfeeding was seen primarily as a nutritive transaction, the relational aspects of breastfeeding were of crucial importance. Parents and staff described the impact of the nipple shield on breastfeeding as a relational activity from three aspects.

#### ‘Good enough’ breasts

Mothers described that the nipple shield was used to stimulate the infant to suck more vigorously. The nipple on the nipple shield was harder, longer and sometimes bigger than their own nipple and therefore triggered the infant to suck in a “better way”. Although no mothers referred to staff telling them that their nipples were not good enough, a suggested usage of a nipple shield implicitly indicated that their breast (s) and breastfeeding could be improved by a nipple shield. One of the nurses described why she very rarely suggested using a nipple shield:

Why use it when it’s equally good without? All babies mature at different paces. Much of this is about our attitudes. I can say that “Yes, you can use a nipple shield sometimes as a temporary remedy or during the whole breastfeeding period. It’s different for different mothers and babies.” And then I talk about how it can be and what babies are capable of. I often say “You have perfect breasts” because really, there are only perfect breasts. Babies can suck on a flat surface so it’s not a hindrance if the nipple doesn’t stick out like a teat. I know that some think that you should use it early, but I think differently. Mothers get a feel that their breasts are not good enough.

#### Something in between

When using a nipple shield there is an evident “in between” effect. Mothers who had not used a nipple shield previously (i.e. when breastfeeding their older children) all referred to the negative aspect of having something in between their breasts and their infant. One mother with twins born in the 32^nd^ gestational week described how she was introduced to a nipple shield and her experiences:

I had just breastfed a few times. She [a nurse] looked and said “she has a bad latch-on”. I didn’t think it was that bad. They [staff] went and got a nipple shield. It wasn’t a suggestion; she just got it as it was the most natural thing to have. I try to remove it when they are hungry but not angry. It’s greasy, you wash them and then it’s goo in them. The breasts feel so clean. And it’s breastfeeding that I have longed for. You don’t feel that close. It’s a sad feeling, it’s a thing. It’s something in between us.

Mothers described that the nipple shield felt “unnatural”, and that it felt more natural without. Mothers also emphasized that it was “plastic”. One of the mothers stated:

I tried it because the mother next to me tried it because her daughter slipped off the nipple. So I thought it would suit us but it didn’t. It was a new taste sensation for her [daughter], the plastic, and she didn’t look content at all.

#### Tranquil moment

Some of the mothers described how the nipple shield helped their infants to latch on. Mothers were grateful for the nipple shield and liked it because it gave them confidence because the infant sucked and got milk:—“Without it, it’s vigorous sucking and he gets annoyed because he doesn’t get a grip and falls asleep right on. With the nipple shield it’s calmer and a nicer rhythm.” Another mother who had experienced that the infant had been sliding across the breast and had had difficulties with the latch because the nipple was too soft now used a nipple shield:—“To use a nipple shield means that they know what to do and that is more pleasant, they eat and swallow.” During one of the observations, a mother and a father were in a single room with their 2 weeks old son born in the 29^th^ week, describing the difficulties in breastfeeding the previous child born in the 31^st^ week. The mother had tried a lot and eventually a nipple shield:

Mother: Dad thought the nipple shield was messy but I thought it was security. This time I tried without it but he just sucked a few times and then lost the nipple. Then dad fetched one [nipple shield] and he got half of the assigned milk volume straight away [the son was weighed before and after breastfeeding]. With a nipple shield you park it in the mouth.

Dad: Now I think it’s [nipple shield] great.

For most mothers the nipple shield was experienced as a hindrance for a tranquil and pleasurable breastfeeding moment. Mothers and staff described the difficulties to attach the nipple shield on to the breast. One of the nurses said:—“I’m not a supporter of nipple shields. It’s really difficult to make it stay attached. There is often a gap [points south-east on an imagined shield]”. Mothers often stated that they needed two hands or sometimes help to put it on as they were holding their baby. The nipple shield kept loosening from the breast, which made the breast all messy and sticky due to leaking milk and the infant got wet.

One couple of parents of a son born in the 31^st^ week were observed and interviewed on 15 occasions, the first one when their son was 1 week old and the last one on the day of discharge. In the 36^th^ week the mother was suggested to use a nipple shield on her left breast, which the son had never been able to suck at. The nipple on that breast was inverted but with the nipple shield he started to suck. In the 38^th^ week, the mother felt stressed and anxious and described that the only thing her son did on the breast with the nipple shield was to suck some milk into the nipple but nothing more. Furthermore, as described by a nurse:—“the son rejects the breast where she doesn’t use a nipple shield”. Hence, the mother started to use nipple shields on both breasts. During a 2-h long observation (07.50–10.50), it was evident how difficult and stressful breastfeeding could be for the mother and father:

*Excerpt from field notes:*

Mother is sitting in a reclining chair reading the newspaper and is getting ready to breastfeed. The father is standing by the washbasin cleaning the nipple shield. The mother lifts up her son from the cot and cuddles him.

Mother [screaming fairly loud]: Now you have to help me! Mum needs help with the nipple shield!

Dad hurries and she places the nipple shield onto her breast.

Mother: I’m not so keen on the nipple shield. It’s so flipping difficult. You should attach it properly and he needs to be in a good position and get it right. And I get so stressed. It’s great if he gets it in to his mouth and sucks.

The nipple shield falls off as soon as the son starts to suck. She places the son in her lap and attaches the nipple shield again, while the son is screaming. She starts to breastfeed again. After about 10 min she takes him off the breast and gives the nipple shield to the dad who cleans it. She shouts to the dad to hurry up and when he gives her the nipple shield she attaches it to the other breast and starts to breastfeed. After 5 min she takes him off the breast. The father checks the position of the tube by a syringe, which he is supposed to aspirates some milk in. He states that there is “nothing” and asks a nurse to check who as well find no milk. Although encouraging words from the nurse that “it does not mean that he has not received any milk by breastfeeding”, the mother walks out disappointed to express milk by a pump.

Two days later the mother had stopped using the nipple shield and breastfed her son on her right breast and expressed on her left breast and was discharged.

#### Progression

The opinions about the advantages/disadvantages of using nipple shields when progressing towards breastfeeding were divergent. Conflicting information, advice and support from staff in relation to nipple shields made parents frustrated, insecure and weary:—“They [staff] say different things about the plastic thing. Some say it is ok to use it. Others say do not - absolutely do not use it! Then you do not know.” Another mother said:—“Yes we used it then, but then another nurse came and said that we should absolutely not use it, it causes a different sucking technique, precisely like that when bottle feeding.” Parents and staff described three major rationales for usage of nipple shields, described in the following sections.

#### Learning quicker

In one of the Swedish units, nipple shields were introduced to mothers very soon after breastfeeding had been initiated. It was experienced by mothers that nipple shields were part of the norm. During one observation, a mother with twins born in the 28^th^ week who had been on ventilators and a CPAP intermittently for months was advised to use a nipple shield. The mother had only had one of her infants at the breast once but the nurse described breastfeeding as a process that started with a nipple shield. One mother with a 1-week old son born in the 32^nd^ week had been provided with a nipple shield 2 days after birth:

They gave me this [nipple shield] and it stimulates the hard palate. In the beginning it came off and it was hard for him to accept it. On day 3 and 4 I tried without it and he sucked then as well. To me it feels more natural without. But from the unit they want me to have it.

The rationale for introducing the nipple shield early on was that infants “learn quicker” to breastfeed. During one observation with a multiparous mother of a son born in the 27^th^ week, now being 10 weeks old, she said:

Mother: I had a nipple shield with my older child ‘cause then I had an ulcer on the nipple. Some staff are so pushy to use it. Before [birth] I didn’t think I would use one unless I had problems. But now they want mothers to use them right from the start. They should learn quicker because it stimulates the palate and then it stimulates the sucking.

Researcher: What do you think?

Mother: I think it’s very individual. Maybe it takes another week. It is better that he can suck and lick as he wants it. I’m not going to prize open his jaws.

In the other Swedish unit, nipple shields were introduced later on. The rationale for a later introduction was described by one of the nurses:—“You should not give a premature infant on a nipple shield too soon. It feels wrong. You have to wait until they are mature enough, so you do not give it to them because of the immaturity itself. Give them time.”

#### Short term solution

For almost all parents and staff the nipple shield was regarded as a temporary device; a device that would be removed “after some time”. However, some nurses described that the negative aspect of using a nipple shield was the difficulty in trying to stop using it as the infant had become used to it. The benefits of using a nipple shield were that it helped the infant to get a good latch-on and that it triggered sucking. Other reasons for using the nipple shield as a short term solution was that the infant had a “small mouth”, that the “breasts were big” or that the nipples were “too flat”. Mothers described that if and when they used a nipple shield it was only a temporary solution and that they would try to not use it as soon as they could: “It can be good short term, but difficult to have for a longer time.” Several nurses did not think that the nipple shield solved any problem and questioned the usage. One of the English nurses stated:—“I am not very happy about using nipple shields and I don’t think they cure problems. I think problems can be cured without them and we need to work around them.” Thus, parents and staff narrated advantages and disadvantages of using nipple shields. However, some believed that the advantages out-weighed the disadvantages and vice versa. One mother with twins described her decision making:

When I don’t have it, they are laying there searching and if they find it they suck a few times and then become tired. It’s instinct. With a nipple shield they know what to do, they eat and swallow. You have to prioritize; the most important thing is that they eat.

#### Rescue remedy

Often, nipple shields were used as a rescue remedy for mothers who might otherwise decide to discontinue breastfeeding if things did not progress. One mother said that the nipple shield helped breastfeeding enabling women and their babies to go home earlier. She felt that a staff had “rescued” them by giving them the nipple shield. One nurse described her views:

If I’m not going to work for a week and I think that a mother would breastfeed exclusively by tomorrow if she gets a nipple shield, I feel that I have to save them from a failed breastfeeding, because I don’t know what will happen if I’m not here. Someone might suggest a bottle. Then it’s better if I give her a nipple shield and with a nipple shield you often get a revolutionary rapid result. If a mother is sitting there with an infant in week 33 who shows rooting but just sucks a few times, then it’s easy to wean. She becomes hesitant to breastfeed because she doesn’t get a response. It gives the mother the response she needs when the infant sucks. And then I can walk out of here without anxiety. If we were to take the nipple shields away, a lot of mothers would start bottle feeding instead.

Some of the mothers described that nipple shields were a step previous to bottle feeding:—“It is perhaps the first step I would take before I would give the bottle.” Another mother described the following:

There are cups to use for feeding and there are nipple shields but there are no bottles to be seen. Bottles are really forbidden. And really, there shouldn’t be such a difference. I know that the babies shouldn’t get used to a bottle because then they can’t suck on the breast. But it can’t be that much of a difference between sucking on a nipple shield or a breast.

Nipple shields were also seen as a rescue remedy for staff; as a way out when they had nothing more to suggest or when they lost patience with mothers attempting breastfeeding. One of the nurses said:—”There are staff that easily give a nipple shield. It’s so easy to give one and then they have done something. Because you don’t have the patience. If I lose patience I ask xxx [name of a nurse with much experience].

## Discussion

The aim of this paper was to describe experiences and perceptions of nipple shields by staff and parents in NUs in Sweden and England. As the use of nipple shields has become unusual in the UK, the data reported here mainly come from Swedish NUs where nipple shields are used to facilitate the preterm infant’s latch on to the breast. This shows two different cultures with regard to breastfeeding support in general [12] and usage of nipple shields in particular. In England, nipple shields were not advocated, a subsequent effect of the implementation of the UNICEF UK Baby Friendly Initiative, whereas Sweden seemed to have a more positive or liberal view on nipple shields.

It became clear from the data that the nipple shield was used as a liminal activity to bridge a gap between uncoordinated sucking and more coordinated sucking. In terms of the relational aspects of breastfeeding this was felt by staff and mothers to potentially undermine mother’s perceptions of their own capabilities in terms of the adequacy of their breasts and breastfeeding. In other situations, the shield was perceived as a physical barrier between the mother and infant. However, for some mothers, when the sucking improved, the shield enabled them to have some quiet time with their infant. These influences upon the relationship between the mother and infant need to be taken carefully into account, given the importance of the relational aspect of breastfeeding [16].

The nipple shield was perceived to assist ‘progression’ by some staff and mothers. This included a sense that the infant would establish breastfeeding, in some cases, earlier and, in others, more quickly. However, this was only seen as a short term solution and in some cases, it was seen as a hindrance to progression. In some situation staff used the nipple shield as a rescue in a situation which otherwise may have led to early cessation of breastfeeding. Given the existing evidence on the increased risk for early cessation of exclusive breastfeeding when using a nipple shield [8, 10], it is important that the potential negative effects of long-term use is communicated to mothers and that knowledge is transferred on how to wean from a nipple shield, if it is felt necessary to introduce the nipple shield in the first place. A nipple shield should hence only be used after the mother has received qualified breastfeeding support and after substantial trying without a nipple shield. As a part of this support it is very important that the mother and baby’s own particular needs are taken into account on an ongoing basis and that the imperative to establish breastfeeding should not take precedence over the relational aspect as is so often the case in term infants [17] and preterm infants [18]. It is also crucial that the mother receives continuity of care and regular assessment of the situation based on a person-centred assessment [19]. In summary, we need to emphasise the importance of the ‘relational’ whilst understanding the need for ‘progression’. Holding these in balance may be the key to appropriate use of the nipple shield.

It needs to be emphasised that use of the nipple shield was not the primary focus of the original research. Had this been the case more observation and interview probing may have taken place related to this specific issue. Ethnographic research takes place ‘in the field’, so the observations and experiences can be made in their actual context. By ‘participating’ in people’s lives for several months, the studying of both the explicit (the more obvious) and the tacit (the hidden) cultural knowledge are enhanced [12]. This study was undertaken during a period of 11 months and conducted in an overt manner, in which the first author used a moderate level of participation [14]. Although the duration of time spent in NUs is extensive, the presence of researcher may have influenced the behaviour of the people who are studied. However, over time this effect appeared to lessen as they habituated to the presence of the researcher [20].

A potential limitation of the study is that the first author who conducted all observations and interviews had worked in neonatal care for more than 10 years and was a native Swede bringing some familiarity and preconceptions [20]. In order to enhance credibility, i.e. whether or not the research findings represent a credible conceptual interpretation of the data and thereby trustworthiness [21], field notes and the field-work diary were discussed between the first and last author who is English and less immersed in neonatal care [22].

One challenge in research is that of presenting the perspectives of others. The researcher has a power to determine which parts of that data are described and how they are presented. With four researchers doing analyses, we believe that our findings represent what was described by parents and staff and not a “skewed” presentation.

Regarding *transferability*, it needs to be recognized that policies and practices are dynamic and changing according to research evidence and expert opinions. Furthermore, as shown, different countries and different NUs may have very different approaches to nipple shields. Hence, the findings from this study should be interpreted and assessed from different cultural perspectives.

## Conclusions

This study illustrates the experiences and perceptions of using a nipple shield in parents and staff in a predominantly Swedish context. The findings show that mothers and staff experienced both negative and positive aspects of using a nipple shield. A nipple shield undermined mothers’ self-confidence and was a barrier to a ‘closer’ and tranquil breastfeeding but was also regarded as a short-term solution and a rescue. Hence, it is very important that the mother and baby’s own particular needs are taken into account, in a person-centred way and on an ongoing basis, and that the imperative to establish breastfeeding should not take precedence over the relational aspect. This is particularly crucial in the case of mothers with a preterm infant as they are inevitably more vulnerable.

In summary, we need to emphasise the importance of the ‘relational’ whilst understanding the need, in some situations, for support with short-term ‘progression’. Holding these in balance may be the key to appropriate use of the nipple shield.

## Abbreviations

CPAP: Continuous positive airway pressure
GW: Gestational week
NU: Neonatal unit
